# Economic impacts of illness in older workers: quantifying the impact of illness on income, tax revenue and government spending

**DOI:** 10.1186/1471-2458-11-418

**Published:** 2011-06-01

**Authors:** Deborah J Schofield, Rupendra N Shrestha, Richard Percival, Megan E Passey, Simon J Kelly, Emily J Callander

**Affiliations:** 1 NHMRC Clinical Trial Centre and Sydney School of Public Health, University of Sydney, Sydney NSW Australia; 2 NHMRC Clinical Trial Centre, University of Sydney, Sydney NSW Australia; 3 National Centre for Social and Economic Modelling, University of Canberra, Canberra, ACT, Australia; 4 University Centre for Rural Health (North Coast), Sydney School of Public Health, University of Sydney, Lismore NSW Australia

## Abstract

**Background:**

Long term illness has far reaching impacts on individuals, and also places a large burden upon government. This paper quantifies the indirect economic impacts of illness related early retirement on individuals and government in Australia in 2009.

**Methods:**

The output data from a microsimulation model, Health&WealthMOD, was analysed. Health&WealthMOD is representative of the 45 to 64 year old Australian population in 2009. The average weekly total income, total government support payments, and total taxation revenue paid, for individuals who are employment full-time, employed part-time and not in the labour force due to ill health was quantified.

**Results:**

It was found that persons out of the labour force due to illness had significantly lower incomes ($218 per week as opposed to $1167 per week for those employed full-time), received significantly higher transfer payments, and paid significantly less tax than those employed full-time or part-time. This results in an annual national loss of income of over $17 billion, an annual national increase of $1.5 billion in spending on government support payments, and an annual loss of $2.1 billion in taxation revenue.

**Conclusions:**

Illness related early retirement has significant economic impacts on both the individual and on governments as a result of lost income, lost taxation revenue and increased government support payments. This paper has quantified the extent of these impacts for Australia.

## Background

Chronic disease has emerged as the major health issue of the 21^th ^century with 60% of deaths worldwide (87% of deaths in high income countries) attributable to chronic conditions [1,2]. In addition to the personal suffering born by patients with a long-term health condition, numerous studies have argued that poor health may also exclude people from the labour force and thus prevent them from obtaining sufficient economic resources [3,4].

Illness related early retirement has been identified as having significant economic impacts in virtually all OECD countries [5]. The economic costs to society come in a variety of forms, and include lost income to individuals and lost tax revenue and increased welfare expenditure for governments [6].

Internationally, some research has been undertaken to assess the economic impacts of various individual diseases [2,7-10]. However, these studies have generally focused only on loss of income and exclude, for example, reductions in occupational based pension plans and reductions in taxation revenue from earned income. In addition, previous research has not examined disaggregated individual outcomes but rather only aggregated outcomes.

This paper provides estimates of some of the indirect costs to individuals and governments that are associated with illness-related early retirement, using the Australian population aged 45-64 as an example. The costs of early retirement have been specifically recognised by the Australian government [11], where it is estimated that 58% of men and 26% of women who retire from full-time work early (before the age of 55 years, the age when Australian citizens can access preserved superannuation) do so because of ill health [12]. This paper builds on earlier work on the impact of chronic disease on labour force participation [3] and utilises Health&WealthMOD-a purpose built, up-to-date microsimulation model of health, income, tax and transfer payments (payments made through the social security system) for persons aged 45 to 64 years. This paper will estimate the amount of income received, amount of taxation paid, and value of transfer payments received by individuals who have retired early due to ill health, compared to other groups in society. It will also provide a national estimate of the amount of lost income, and taxation revenue, and additional transfer payments made that could be attributed to illness related early retirement in 45 to 64 year old Australians. As such, the paper takes both a governmental perspective (by estimating additional transfer payments and lost taxation), and an individual perspective (by estimating the costs of lower personal incomes).

## Methods

### Data

**Figure 1:**
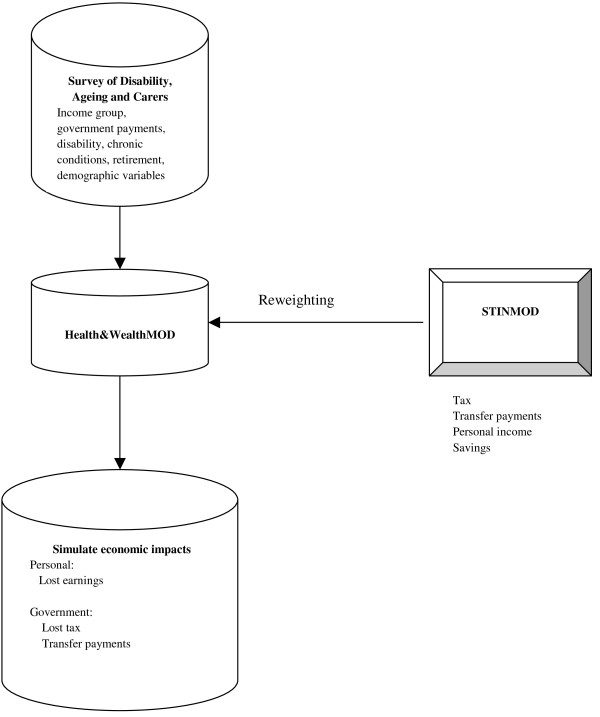
Estimates of lost income, tax and benefits in Health&WealthMOD.

We analysed the output dataset of a microsimulation model, Health&WealthMOD, which is Australia's first microsimulation model of health and disability, the associated impacts on labour force participation, personal income, and government revenue and expenditure. It was specifically designed to measure the economic impacts of ill health on Australian workers aged 45 to 64 years. The process by which Health&WealthMOD was built is outlined in Figure 1 and detailed below.

The base population of Health&WealthMOD was unit record data extracted from the Survey of Disability, Ageing and Carers (SDAC) conducted by the Australian Bureau of Statistics in 2003 [13]. The individual level information collected in the survey allows the modelling of a range of impacts that disability and specific illnesses can have on labour force participation and income, and the capturing of variation in potential earnings and capacity to save. From this dataset, individual records were extracted for those aged 45-64 years and for other persons in their family (income unit). The details extracted for each individual in the base population included demographic variables (for example, age, sex, family type, state of residence, and ethnic background), socioeconomic variables (level and field of education, income, benefits received), labour force variables (labour force participation, employment restrictions, retirement), and health and disability variables (chronic conditions, health status, type and extent of disability, support and care required).

Using a separate microsimulation model--STINMOD--additional economic information such as individual income, transfer payments, tax liabilities and the value of wealth in different types of assets, which were not available in the SDAC dataset, were imputed onto the base data (Table 1 shows the variables that were taken from the SDAC and STINMOD). The data were up-rated to reflect 2009 currency and to account for the demographic, labour force, earnings growth and other changes that had occurred between 2003 and 2009. STINMOD is Australia's leading model of income tax and government benefits,[14] and is maintained and developed for the Australian Government by the National Centre for Social and Economic Modelling. It is routinely used by government departments for assessing the distributional and revenue implications of tax and cash transfer reforms. The model operates at the 'micro' level of families and individuals, and uses Australian Bureau of Statistics Survey of Income and Housing Costs and Amenities records as the base population.

**Table 1:** Source of variables in Health&WealthMOD

SDAC	STINMOD
Age	Age

Sex	Sex

Income unit type	Income unit type

Type of government pension received	Type of government pension received

Income quintile	Income quintile

Labour force status	Labour force status

Hours worked per week	Hours worked per week

Highest education attainment	Highest education attainment

Long term health condition	

Reason not in the labour force	

	Continuous income

	Taxation paid

	Continuous transfer income

	Values of different asset types

The original SDAC data was weighted by the Australian Bureau of Statistics to broad population variables such as age and sex. However, in building a microsimulation model to cost the financial impact of illness, it is important to ensure the correct weighting for any crucial policy-related sub-populations. In this case it was important to ensure that the number and age/sex distribution of sickness and disability support pension beneficiaries was accurate. Therefore the sickness and disability support pension beneficiary status were also reweighted. The data were reweighted to represent the 2009 population.

It is not possible to exactly match individuals between STINMOD and the SDAC. As both are based on survey information there are few respondents in common on both data sources. Further, the data was collected at different points in time, meaning that even for the few individuals that may be in common, some variables will no longer be the same between the SDAC and the surveys underpinning STINMOD, or will have changed due to the ageing and uprating process which ages STINMOD data to 2009 e.g. age, income, labour force status and income unit type. Furthermore, for privacy reasons exact matching between Australian Bureau of Statistics surveys is prohibited. As a result of these constraints, income and wealth information was imputed onto the base population of Health&WealthMOD by synthetically identifying persons with similar characteristics on STINMOD and "donating" their income and wealth information onto Health&WealthMOD [15].

Eight variables: sex (2 groups), income unit type (4 groups), type of government pension/support (3 groups), income quintile (5 groups), age group (4 groups), labour force status (4 groups), hours worked per week (5 groups) and highest educational qualification (2 groups), that were common to both datasets and strongly related to income were chosen as matching variables for synthetic matching.

In this paper, we used Health&WealthMOD to estimate loss of income, taxation revenue and additional government benefit payouts attributed to illness related early retirement. The other major features of this microsimulation model are that it can be used to estimate the accumulated loss of wealth at one point in time, and to project the loss of savings associated with illness related to early retirement from the age of retirement through to the traditional retirement age of 65 years. Health&WealthMOD can also be used to model the economic gains, in terms of increased personal incomes and taxation revenue and decrease in government benefit payments, as a result of certain health interventions which reduces the incidence of chronic diseases and the associated number of people who could remain in the labour force if they were not ill.

### Statistical methods

A multiple linear regression model of the log of weekly income was used to analyse the differences between weekly incomes of people in the labour force (full-time and part-time) and people not in the labour force due to ill health. Analyses were repeated for weekly transfer income and weekly tax liability. Co-variates: age group, sex and highest education were adjusted for in all regression models. Regression analysis was undertaken on log-transformed data in order to satisfy the assumptions of linear regression analysis, and regression diagnostics confirmed that the assumptions were reasonably satisfied. As data analyses were undertaken on a log-scale, geometric means were presented in the results, and thus "average" refers to the geometric means in this paper. The analyses were undertaken using SAS V9.1 (SAS Institute Inc., Cary, NC, USA). All statistical tests were two sided with the significance level set at 5%.

Ethical approval was not required, but the research conformed to research conformed to the Helsinki Declaration http://www.wma.net/e/policy/b3.htm, and to local Australian legislation.

## Results

Among 9,198 people aged between 45 and 64 years surveyed for the Survey of Disability, Ageing and Carers, 4,266 (46%) were in full-time employment, 1,738 (19%) were in part-time employment and 661 (7%) were not in the labour force due to ill health. Once weighted, this data represented 2,566,200 (48.5%) full-time and 975,300 (18.4%) part-time working individuals, and around 415,700 (7.9%) individuals not in the labour force due to ill health in the Australian population aged between 45 and 64 years in the year 2009. Persons out of the labour force due to ill health had an average weekly income (including transfer income) of $217.8 (all values are 2009 Australian dollars), which is less than one-quarter of the income of those employed full-time ($1167.0) and less than half of the income of people in this age group who were employed part-time (Table 2).

**Table 2:** Geometric means of weekly income, transfer payments and tax liability by labour force status for the Australian population aged 45-64 years, 2009

Labour force status	Weekly income	Weekly transfer income	Weekly tax liability (includes Medicare levy)
Employed full-time	1167.0	0.2	166.3
Employed part-time	482.9	0.9	8.9
Not in labour force due to ill health	217.8	74.2	0.0

Note: a)Transfer income includes family payment; b) Negative incomes were excluded from the analysis.

People who were not in the labour force due to ill health received a significantly higher amount of transfer income than people who were employed part-time or full-time (Table 2). The average amount of weekly tax liability of those who were not in the labour force due to ill health was far lower than the tax paid by those who were employed full time and employed part-time.

After adjusting for age, sex and education, people who were out of the labour force due to ill health had 78.9% (95%CI:-80.5 to-77.2) lower average weekly incomes than those who were employed full time (Table 3). Government spending on transfer payments to those out of the labour force due to ill health was significantly higher than the spending on transfer payments to those who were employed. People who were out of the labour force due to ill health also paid 99.94% (95% CI:-99.95 to-99.93) less tax per week than those who were employed full time.

**Table 3:** Differences in average weekly income, transfer payments and tax liability between labour force status, adjusted for age group, sex and education, for the Australian population aged 45-64 years, 2009

Labour force status	Income			Transfer income			Tax liability (includes Medicare levy)		
	
	% difference	95% CI	p-value	% difference	95% CI	p-value	% difference	95% CI	p-value
Employed full-time		*Reference*			*Reference*			*Reference*	
Employed part-time	-52.4	(-55.2,-49.5)	<.0001	121.6	(97.9, 148.2)	<.0001	-93.1	(-94.1,-92.1)	<.0001
Not in labour force due to ill health	-78.9	(-80.5,-77.2)	<.0001	14503.9	(12468.3, 16869.3)	<.0001	-99.94	(-99.95,-99.93)	<.0001

The 7.9% of the total Australian population aged between 45 and 64 years who reported that they were out of the labour force due to their ill health, resulted in a national annual loss of personal income of about $18 billion, total lost tax revenue of $1.5 billion, and increase in social security payments of $2.1 billion (Table 4).

**Table 4:** National annual impact of persons not in the labour force due to ill health (adjusted for age, sex and education) for the Australian population aged 45-64 years, 2009

	Income	Transfer Payments	Taxation Revenue
Not in labour force due to ill health	17,989,175,000	1,468,007,000	2,052,384,000

Note: Based on the differences between persons not in the labour force due to ill health and the weighted average of persons employed full time and part time

## Discussion

Based on outcomes from Health&WealthMOD, persons out of the labour force due to ill health are estimated to have significantly lower average weekly incomes than those that are in the labour force. Additionally, the modelling shows that those who are out of the labour force due to ill health receive higher average weekly transfer payments and pay less tax.

This study does have some limitations to consider. In particular, the findings are based upon respondents' self-reported data. Although self-report health and economic status are regarded as valid measures [16,17], the potential for bias in the results cannot be excluded.

Brazenor (2002) found that disabled males earn 83 per cent, and disabled females earn 76 per cent, of the incomes of their non-disabled counterparts. This is consistent with the findings of this study which has found that individuals with no condition have higher average weekly income than those who do have a chronic health condition. Brazenor (2002) also found that the impact of ill health upon earnings varied with the type of condition [18]. However, Brazenor (2002) focused only on the impact of illness or disability on labour market earnings, and did not take into account the reduced comparative income from all sources when individuals are forced to retire from the labour force early due to their illness. This study analysed the total income from all sources which included not only wages, but also all other private sources, and transfer payments.

Retiring due to illness is more common among those aged over 44. Wilkins (2003) found that the impact of a disability on employment increased with age, with those aged over 44 having lower labour force participation than younger people with a disability [19]. Thus, including the cost of lost income due to early retirement as a result of illness is particularly important when considering heath impacts in the 45-64 year age group.

The loss of income due to illness identified in this study may also impact upon individuals' living standards. Himmelstein *et al*. (2005) found that in the United States one quarter of bankruptcies were attributed to chronic illness; and 35 per cent of bankrupts were no longer employed due to their own, or a family members' illness. Additionally, lost income was associated with inadequate finances for basic services and medical care [20].

This paper highlights the cost of illness from an individual and governmental perspective. It has shown how people of an older working age who have fallen ill and have been forced to leave the labour force because of their illness now face the prospect of poorer financial living standards due to their lower incomes. This is in addition to the limitations and physical and mental hardship that will be caused by the illness. Other studies have shown that individuals who have retired from the workforce due to ill health will not only have lower incomes, but also lower amounts of wealth, and by the time they reach the traditional retirement age of 65 years they will also have a smaller amount of savings with which to support themselves [21-23]. The Australian government has made efforts to assist disabled workers to return to the labour force, and strongly supports older and disabled workers returning or continuing in the workforce [24-27]. However, for those who are not able to work, their personal living standards are likely to be affected due to their lower economic resources compared to those in the labour force.

This study estimated that the increased amount of transfer payments received by individuals who are out of the labour force due to their ill health is $1.5 billion annually. This quantified the increased burden that transfer payments have on Australian governments, which has been recognised in other studies. Cai and Gregory (2002) found that from 1972-2002 there has been an annual growth of over 5 per cent in the number of people receiving a Disability Support Pension in Australia [28], similar findings were also reported by Wilkins (2003). Wilkins (2003) also found that the age of male Disability Support Pension recipients was decreasing [19]. This indicates that in Australia more people are retiring early due to illness and that the illness related early retirement age is decreasing.

Other authors have noted that a decline in workforce participation as the result of early retirement will also contribute to lost taxation revenue [29,30]. This study demonstrated that this annual lost taxation revenue due to ill health alone is $2.1 billion. This reduction in taxation revenue will place a strain on government budgets in meeting the costs of increased numbers of people receiving disability pensions and the reliance upon public health services produced by illness related early retirement [17,31].

Cai and Kalb (2004) and Kohli and Rein (1991) both discuss the strain that early retirement places upon government budgets as income from taxation declines and spending on benefits increase [17,31]. The *Economic Implications of an Ageing Australia *report states that the labour force participation rate is expected to fall by seven per cent by 2044-45, as low fertility rates have resulted in a deficit of younger workers to replace those who retire [30]. This makes the participation of those who have not yet reached retirement age even more important. The *Intergenerational Report 2007 *found that workers aged over 55 already have a relatively low level of labour force participation in Australia compared with other OECD countries, and that this will become increasingly significant in the future as the number of people aged between 55 and 64 is anticipated to grow by 50 per cent by 2044-45, making it the fastest growing group of working aged people [29]. With the ageing population, retaining these older workers will be particularly important to maintain workforce participation which is seen as one of the best ways of maintaining economic growth [29,32]-it is estimated that a five per cent increase in labour force participation by 2046-47 could lead to a five per cent increase in GDP by 2046-47 [4]; and providing revenue for government spending to support the ageing population [31].

This paper has highlighted the costs associated with illness related early retirement to both individuals and to government. This differs from the friction cost method, which argues that after a short period people who leave the labour force due to ill health will be replaced by other workers (either people who were previously unemployed or the relocation of existing employees) and that productivity losses associated with health are limited to the period needed to replace an ill worker [33]. However, for individuals who leave the workforce permanently due to ill health the economic impacts will continue. In addition, Australia has a very low unemployment rate (5% in January 2011) [34] and significant labour shortages in some industries [35,36], thus the high number of people out of the labour force due to ill health is a significant constraint to economic growth. This has been highlighted by the Australian Treasury who aim to make Australia's financial position more sustainable by promoting productivity, population growth and labour force participation [37,38].

Investment in preventive health measures is one way of overcoming the detrimental impacts that ill health has on workforce participation [39]. The findings of this study align with the Australian Government's health platform, which recognises that chronic disease prevention can increase labour force participation and ensure future government revenue is sufficient to fund health care for an ageing population [40,41]. A preventive health approach aims to reduce debilitating illness which often leads to early retirement. Such outcomes will maintain, and possibly increase workforce participation [42,43], thereby helping to maintain economic growth through maintaining human capital in production [29,44].

## Conclusions

In the past, policy has focused upon economic incentives to defer retirement [39,45]. However, as ill health is a primary barrier to workforce participation in older Australians, economic incentives alone may not be able to increase participation if the underlying health conditions are not addressed. Investment in improvements in health is potentially an important way of improving national living standards.

## Competing interests

The authors declare that they have no competing interests.

## Authors' contributions

DS conceived and lead the study. RS and RP undertook data analysis and the creation of Health&WealthMOD. MP and SK contributed to the research design. RS and EC prepared the original manuscript. All authors contributed to the study design and manuscript editing. All authors read and approved the final manuscript.

## Pre-publication history

The pre-publication history for this paper can be accessed here:

http://www.biomedcentral.com/1471-2458/11/418/prepub
